# Neuromodulation as a new avenue for resuscitation in hemorrhagic shock

**DOI:** 10.1186/s42234-019-0033-z

**Published:** 2019-10-24

**Authors:** Keren Powell, Kevin Shah, Caleb Hao, Yi-Chen Wu, Aashish John, Raj K. Narayan, Chunyan Li

**Affiliations:** 1 Translational Brain Research Laboratory, Feinstein Institutes for Medical Research, Manhasset, NY USA; 2 Department of Neurosurgery, Zucker School of Medicine at Hofstra/Northwell, Hempstead, NY USA; 3 Feinstein Institutes for Medical Research, 350 Community Drive, Manhasset, NY 11030 USA

**Keywords:** Hemorrhagic shock, Neuromodulation, Vagus nerve stimulation, Trigeminal nerve stimulation, Phrenic nerve stimulation, Electroacupuncture, Resuscitation, Inflammation, Autonomic nervous system

## Abstract

Hemorrhagic shock (HS), a major cause of early death from trauma, accounts for around 40% of mortality, with 33–56% of these deaths occurring before the patient reaches a medical facility. Intravenous fluid therapy and blood transfusions are the cornerstone of treating HS. However, these options may not be available soon after the injury, resulting in death or a poorer quality of survival. Therefore, new strategies are needed to manage HS patients before they can receive definitive care. Recently, various forms of neuromodulation have been investigated as possible supplementary treatments for HS in the prehospital phase of care. Here, we provide an overview of neuromodulation methods that show promise to treat HS, such as vagus nerve stimulation, electroacupuncture, trigeminal nerve stimulation, and phrenic nerve stimulation and outline their possible mechanisms in the treatment of HS. Although all of these approaches are only validated in the preclinical models of HS and are yet to be translated to clinical settings, they clearly represent a paradigm shift in the way that this deadly condition is managed in the future.

## Background

Hemorrhagic shock (HS) is classified as a type of hypovolemic shock resulting from rapid and significant loss of intravascular volume (Blalock 1940) leading to hemodynamic instability, decreased oxygen delivery and tissue perfusion, cellular hypoxia, organ damage, and if untreated death (Millham 2010). This is distinct from rapid exsanguination, which, while potentially lethal, does not necessarily lead to the development of a shock state. The causes of HS are multivariate, some of which include traumatic injury, upper or lower gastrointestinal bleeding, obstetric and gynecologic bleeding, ruptured aneurysms, and iatrogenic vascular injuries (Cannon 2018). On the whole, it is a significant cause of mortality, accounting for more than 60,000 deaths per year in the US and an estimated 1.9 million deaths worldwide (Cannon 2018). In the US, hemorrhage and HS caused by severe traumatic injury account for one third of related deaths and are the main cause of death in young people and combat individuals (Eastridge et al. 2012; Tisherman et al. 2015). Severe trauma and hemorrhage can be rapidly lethal; more than 50% of trauma-related deaths due to HS occur during the prehospital period, and 93% of post-hospitalization deaths occur within the first 24 h period (Alam 2017; Holcomb et al. 2013). The current standard of care for HS consists of timely hemostasis, volume replacement, and whole blood or blood component therapy (Gann and Drucker 2013; Jacob and Kumar 2014; Nair et al. 2017). In the last 10 years, advances in hemorrhage pathogenesis and treatment have led to only modest improvements in survival, with fundamental issues such as timing and volume of fluid intervention remaining controversial (Kwan et al. 2003). Therefore, there is an unmet and critical need for a novel and effective adjuvant therapy to improve the survival and recovery of individuals that suffer HS.

### Pathophysiology of hemorrhagic shock

Severe blood loss leading to systemic hypoxia results in irreversible lethal damage of tissues and organs. The systemic response to blood loss acts to maintain levels of arterial pressure (AP) necessary to provide adequate perfusion pressure (PP) for sufficient oxygen delivery (Gutierrez et al. 2004; Moore 2014; Schiller et al. 2017). At early stages of hemorrhage, decreased AP and blood volume activate peripheral and central baro- and volumo- receptors and trigger compensatory sympathetic and endocrine mechanisms (Bonnano 2011). Sympathetic activation induces tachycardia and vasoconstriction of the ischemia-tolerant structures, increasing myocardial oxygen consumption. Cerebral and cardiac autoregulation provides sufficient blood supply down to an AP of ~ 60 mmHg, however further blood loss exhausts compensatory mechanisms leading to hypoxia. The brain’s response to hypoxia further activates the sympathetic system as a “last-ditch” response; failure of this response to provide sufficient cerebral PP deepens ischemia. The decrease in myocardial perfusion results in myocardial ischemia, reduced cardiac contractility and output, and further impairment of circulation known as decompensation. A severe drop in PP leads to intravascular blood flow stasis, microvascular thrombosis and activation of immune mechanisms. Multiorgan hypoxia develops, resulting in multiple organ failure and acute respiratory deficiency syndrome leading to irreversible shock (Del Sorbo and Slutsky 2011; Dutton 2007). The core of the systemic response to hemorrhage is directed towards the maintenance of AP and PP; readjustment of the autonomic balance, attenuation of sympathetic hyperactivity paralleled by increase in parasympathetic tone, increase in brain and organs hypoxic tolerance, and augmentation of blood supply to vital organs are crucial for preventing decompensation from hemorrhagic shock.

## Neuromodulation

Neuromodulation, also commonly referred to as electroceuticals or bioelectronic medicine, is a therapeutic technique that involves transferring energy into the nervous system to excite, inhibit, or otherwise modify neural activity (Famm et al. 2013; Gildenberg 2006; Krames et al. 2009). Modulation of neural activity via targeted electrical stimuli may represent a paradigm shift for the treatment of a wide range of diseases (Luan et al. 2014). Various forms of neuromodulation have been investigated as treatments for HS including vagus nerve stimulation (VNS), trigeminal nerve stimulation (TNS), phrenic nerve stimulation (PhNS), and electroacupuncture (EA), each of which targets different stages of HS development (Table 1).

**Table 1:** Novel Neuromodulation Methods of Hemorrhagic Shock

Neuromodulation	Intervention	Animal Species	Observed Effect	Author/Year
Vagus Nerve Stimulation (VNS)	VNS preceding T/HS	Male Sprague-Dawley Rats	Prevention of T/HS-induced gut injury Prevents formation of toxic lymph	Levy et al. 2013
	VNS preceding HS	Male Sprague-Dawley Rats	Decreased gut and lung permeability Decreased pulmonary neutrophil sequestration	Levy et al. 2012
	VNS preceding T/HS	Male Balb/c Mice	Prevention of T/HS-induced changes in gut barrier integrity Decrease in T/HS-induced lung injury Decreased binding of NF-kB	Reys et al. 2013
	VNS following T/HS	Male Sprague-Dawley Rats	Reduction in histologic gut injury Reduction in ML flow	Morishita et al. 2014
	VNS following T/HS	Male Sprague-Dawley Rats	Prevention of changes in ML exosome protein payload Limitation of systemic inflammatory response	Williams et al. 2019
	VNS following HS	Male Sprague-Dawley Rats	Attenuation of peak serum TNF-alpha amounts Inhibition of liver NF-KappaB expression Prevented the development of hypotension	Li et al. 2005
	VNS following HS	Male Wistar Rats	Increase in survival time Reversion of hypotension Modulation of inflammatory response	Guarini et al. 2003
	VNS during resuscitation	Male Wistar Rats	Decreased inflammatory response to HS Improved coagulation	Rezende-Neto et al. 2014
Electroacupuncture (EA)	EA at ST36 + Delayed Fluid Replacement	Male Sprague-Dawley Rats	Inhibition of plasma adrenal gland concentration increase Enhances the therapeutic effect of DFR	Wang et al. 2018
	EA at ST36 + Delayed Fluid Replacement	Male Sprague-Dawley Rats	Improves blood pressure Raises early survival rate of HS rats Maintains intestinal barrier function Improves the degree of intestinal ischemia	Shi et al. 2013
	EA at ST36 following HS	Male Sprague-Dawley Rats	Attenuation of systemic inflammatory response Intestinal barrier protection Organ function improvement Survival rate improvement	Du et al. 2013
	EA at ST36 + Delayed Fluid Replacement	Male Sprague-Dawley Rats	Protective effects upon hepatic ischemic injury following HS	Zhong et al. 2012
	EA at ST36 following HS	Male Mongrel Dogs	Improvement of hemodynamics Attenuation of cytokine and lactate blood concentration in HS.	Suo et al. 2011
Trigeminal Nerve Stimulation (TNS)	Percutaneous TNS following HS	Male Sprague-Dawley Rats	Modulation of both SNS and PNS Pressor response Improved cerebral perfusion Decreased inflammation	Li et al. 2019
Phrenic Nerve Stimulation (PhNS)	PhNS in combination with ITV	Female Swine	Improvement in cardiac preload and key hemodynamic variables	Samniah et al. 2003

### Vagus nerve stimulation

The vagus nerve is the 10th cranial nerve and the chief parasympathetic branch of the autonomic nervous system (ANS), with an important role in communication with different organs (Borovikova et al. 2000; Breit et al. 2018). Due to its well-known anti-inflammatory properties (Czura et al. 2010; Li et al. 2005; Rezende-Neto et al. 2014; Samniah et al. 2003; Suo et al. 2011; Shi et al. 2013; Tracey 2007; Wang et al. 2018), it is the most widely investigated neuromodulation approach for HS, as HS initiates an injurious inflammatory response (Abraham et al. 1995; Chaudry et al. 1993; Ertel et al. 1994; Karakozis et al. 2000; Schwartz et al. 1995; Tsung et al. 2005; Zingarelli et al. 1994). VNS, known for its anti-inflammatory properties, presents as a promising avenue of study for HS treatment that has been applied to three different phases: pre-hemorrhage, post-hemorrhage, and coagulation improvement.

#### VNS for pre-hemorrhagic phase

The gut plays a key role in the development of intestinal and systemic inflammatory response and gut barrier failure is a major contributor to organ failure following HS (Baker et al. 1988; Deitch et al. 1994; Magnotti et al. 1998). VNS preceding HS has been shown to protect the gut, recover lung permeability, and limit the development of a systemic inflammatory state. VNS prevented a 10-fold increase in gut permeability following HS, as well as mitigating lung injury, as reflected in decreased lung permeability to Evans blue dye and in vivo plasma FD4 permeability (Levy et al. 2012; Levy et al. 2013). While Levy et al. indicated that these effects seemed to have no connection to the activity of the spleen, they did not indicate any actual potential pathway, either cholinergic or otherwise. Similar conclusions have been drawn by a study from Reys et al., wherein VNS preceding HS decreased both the activity of myeloperoxidase enzyme and NF-κβ in lung tissue, though there was yet again no pathway determined for VNS’ actions (Reys et al. 2013).

#### VNS for post-hemorrhagic phase

Inflammatory cytokines play a pivotal role in the pathogenesis of HS (Tracey 2007). In a study from Guarini et al., VNS following HS prevented the liver’s inflammatory cascade, blunted NF-β activation, reduced IκBα protein loss from cytoplasm, blunted hepatic TNF-α mRNA expression, protected against the hypotension, and prolonged survival (Guarini et al. 2003). The protective effects of VNS in this model were reversed by administration of a nicotine receptor antagonist (chlorisondamine), indicating that vagus nerve control of cytokine damage is attributable to cholinergic signals transduced intracellularly via nicotinic receptors. In another study, constant VNS significantly reduced peak serum TNF-α levels, as well as blunting liver NF-κβ activation and decreasing hypotension (Li et al. 2005). These results were tied to the cholinergic anti-inflammatory pathway, which has now become recognized as the major pathway responsible for the anti-inflammatory effects of VNS.

While these two groups explored the systemic anti-inflammatory effects of VNS, other studies explored its effects upon the mesenteric lymph (ML) following trauma/HS (T/HS) (Morishita et al. 2014; Williams et al. 2019). T/HS induced gut injury, increased ML flow, and decreased CD103+ MHC-II+ ML DC, and Foxp3+ MLN T reg cell populations (Morishita et al. 2014). The addition of VNS following T/HS abrogated both gut injury levels and ML flow. A recent study expanded upon these results in search of a distinct exosome phenotype for T/HS, a potential lead for a mechanism for VNS treatment. VNS abrogated the T/HS-induced alterations in ML exosome protein burden, thus suggesting a pathway by which the neuroenteric axis may inhibit the systemic inflammatory response following T/HS (Williams et al. 2019).

#### VNS on activation of coagulation

Coagulopathy is responsible for roughly 50% of trauma associated deaths, and is related to the HS-induced inflammatory response, wherein inflammation is considered a key initiator of trauma associated coagulopathy. The acute hemostatic effect of VNS on coagulation and cytokine production has been investigated in a controlled rat HS model (Rezende-Neto et al. 2014). While there was no significant difference in MAP response, results showed that animals treated with VNS had a significant decrease in IL-1 levels, when compared to both the HS control and fluid resuscitation-only control groups. Furthermore, the rats which underwent VNS showed increased clot firmness and improved clot formation kinetics. Since inflammation can result in both inhibition and exacerbation of the clotting process, the authors hypothesized that VNS produced a more balanced hemostatic response that ultimately resulted in improved coagulation. This inference is further supported by the study from Czura et al. (Czura et al. 2010). In this study, electrical VNS regulated hemostasis, attenuated peripheral hemorrhage, and reduced shed-blood volume in a pig model. While this study did not focus on HS explicitly, the effect of VNS on coagulopathy adds information for a more complete understanding of this concept. Both studies showed that the effects of VNS on bleeding time were independent of effects on heart rate or blood pressure, suggesting that the coagulation activity of blood can be directly modulated by vagal activation.

### Electroacupuncture

Neuromodulation with acupuncture or electroacupuncture (EA) is used by millions of people to control inflammation and reestablish physiological homeostasis (Ulloa et al. 2017), and the use of EA following HS has been examined in some depth. In particular, the effect of EA at ST36 (Zusanli point) has been the focus of numerous experimental efforts (Suo et al. 2011). Often considered to be the main point of effect for gastro-intestinal function, promoting gastrointestinal detoxification, peristalsis, and protection of the mucosal lining, EA represents a promising therapeutic target (Shi et al. 2013). Additionally, EA at ST36 has also been shown to have protective effects on cardiac tissue following HS, through improvement of autonomic nervous function. EA stimulation following HS decreased overexpression of the sympathetic nervous system (SNS), via promotion of acetylcholine release and the resultant activation of the parasympathetic nervous system (PNS) (Wang et al. 2018).

The majority of the pre-clinical work of EA at ST36 focused on protecting the gastro-intestinal region. In a study from Suo et al., the effects of EA on basic hemodynamics, cytokines, and lactate were investigated in mongrel dogs (Suo et al. 2011). Treatment with EA immediately following HS resulted in significant improvements in hemodynamics and attenuation of inflammatory responses. Specifically, gradual increases in MAP, cardiac index, cardiac output, pulmonary wedge arterial pressure, and central venous pressure were observed, as well as decreased levels of serum TNF-α and lactate. Given their results, the authors speculate that ST36 acupuncture may act through activation of the cholinergic anti-inflammatory pathway, as well as possibly, the α7 subunit of nicotinic receptor in order to exert anti-inflammatory effects. Additionally, they propose similarities to vagal electrostimulation through its activation of the cholinergic anti-inflammatory pathway, and suggest it as a clinical alternative to acupuncture. EA at ST36 also further supports its beneficial effects on gut tissue following HS; the amount of diamine oxidase released from the intestinal tissue following HS decreased when the EA is applied which represents valid proof for preservation of intestinal tissue (Shi et al. 2013).

The effects of EA at ST36 to decrease gut barrier dysfunction have also been investigated (Du et al. 2013). In a study by Du et al., a wider range of biological markers, including MAP, interleukin-6, interleukin-10, TNFor-α organ parameters, gut injury score, gut permeability to 4 kDa FITC-dextran, and expression and distribution of tight junction protein ZO-1, as well as simple survival counts, were investigated. Both interleukin-6 and TNF-α levels were reduced after EA, indicating a possible anti-inflammatory effect. This is additionally supported by the fact that gut injury, as measured by levels of damage to the intestinal villus and degree of intestinal permeability, was alleviated with application of EA. In regards to more broad measures of well-being, experimental results showed a significant improvement in MAP following EA, as compared to control groups, and an increase in 12-h survival rates. In another study, the effect of EA on the volume of hepatic blood flow, plasma alanine aminotransferase, and water ratio in rats with delayed fluid replacement following HS was investigated (Zhong et al. 2012). The authors concluded that EA combined with delayed fluid replacement was protective against hepatic ischemia in rats following HS. These results alongside the results from their later study (Du et al. 2013), which includes data from a control group that underwent vagotomy before EA, indicate that EA at ST36, even though not directly stimulating the vagus nerve, does act, at least partially, through the vagal signaling pathway.

### Trigeminal nerve stimulation

The trigeminal nerve (Cranial Nerve V) is the largest cranial nerve. It arises from the Pons and immediately divides bi-laterally at the trigeminal ganglion into three major branches (ophthalmic, maxillary, and mandibular) spread bilaterally along the face (De Giorgio et al., 2011). Trigeminal afferent fibers carry sensory information from the face and project to a range of targets within the brain via the central nervous system (CNS) (Mercante et al. 2017). This then spreads “down” to the autonomic nervous system (ANS), with the potential to modulate the nervous, cardiovascular, and respiratory systems (Buchholz et al. 2017). The ANS is pivotal in regulating the functions of our internal organs and maintaining vital signs and homeostasis (McCorry 2007), and major autonomic instability is a common feature in extreme situations such as hemorrhagic shock (Cooke et al. 2008; Li et al. 2019). The ANS comprises two sets of complementary neuronal circuits, the SNS and the PNS; as hemorrhage ensues the balance of these two systems is disturbed. Unlike the therapeutic mechanism of TNS upon the CNS (Mercante et al. 2018), its effects on the ANS have been widely neglected in the search for treatments and poorly investigated so far. In a recent study from Li et al., TNS appeared to rebalance the ANS in a rodent model of severe hemorrhagic shock, leading to longer survival time following HS (Li et al. 2019). The authors postulated that the beneficial effects of TNS are due to integrated sympathetic and parasympathetic responses, with the SNS maintaining blood pressure and organ perfusion and the PNS attenuating shock-induced inflammatory cascades. Without fluid resuscitation, TNS following severe HS significantly attenuated sympathetic hyperactivity, paralleled by an increase in parasympathetic tone, delayed hemodynamic decompensation, and improved brain perfusion. Furthermore, TNS generated sympathetically mediated low-frequency oscillatory patterns of systemic blood pressure associated with an increased tolerance to central hypovolemia and increased levels of circulating norepinephrine levels. TNS also decreased systemic inflammation compared with the control group. These are novel and impressive differences in physiologic patterns and mortality between the TNS treatment and the control groups during the lethal hemorrhagic insult. However, based on the complex interplay and considerable overlap in intricate neuronal pathways such as trigemino-vagal connection (Chiluwal et al. 2017), further studies are required to refine and confirm the specificity of TNS’s benefit in HS.

### Phrenic nerve

The phrenic nerve innervates the entire diaphragm, and activation of phrenic afferents has been shown to increase ventilation and breathing function in patients with respiratory insufficiency. Phrenic nerve stimulation (PhNS), also known as diaphragm pacing, is electrical stimulation of the phrenic nerve using a surgically implanted device (Khong et al. 2010). In a porcine model of severe hemorrhagic shock, bilateral, transcutaneous PhNS, alongside an inspiratory impedance threshold valve (ITV), improved cardiac preload and vital hemodynamic variables (Samniah et al. 2003). The combination of PhNS and ITV significantly improved right and left ventricular diameters when compared to hypovolemic shock values. Furthermore, the two treatments in conjunction increased transaortic, transpulmonary, and transmitral valve blood flow. PhNS was combined with ITV, in the first place, in order to preclude respiratory gas inflow into the lungs during electrically mediated respiration. This action causes a conversion of the thorax into a pump which draws venous blood from the extremities and splanchnic system into the right side of the heart. Spontaneous gasping respiration, which the action mimics, has been associated with improved outcomes in animal models of cardiac arrest, as well as in clinical patients suffering from cardiac death (Clark et al. 1992). The presented data implies that electrically induced gasping augments cardiac output via increasing venous blood return. This ultimately preserves perfusion pressure and flow to vital organs during HS. Based on the results of this feasibility study, additional investigations with this new technique work are warranted.

## Conclusions

The optimal pre-hospital resuscitative strategy after hemorrhagic shock is controversial. As such, alternative or adjuvant neuromodulation therapies such as VNS, TNS, PhNS, or EA present a potentially fruitful avenue of research for HS resuscitation. Temporizing measures for hemorrhage control through VNS or TNS could be lifesaving, as these neuromodulation methods exploit endogenous compensatory responses that theoretically protect patients from the known hazards of current resuscitative modalities. Attenuation of the systemic immune response through VNS, TNS, or EA could reduce the need for empiric antibiotics and the resulting resistance and antimicrobial treatment failures that are becoming an ever-increasing problem in the fight against sepsis. Furthermore, endogenous neural stimulation by TNS might not only obviate the need for exogenous vasopressors, but also promote fluid sparing, thereby potentially mitigating the complications of aggressive fluid resuscitation, such as dilutional coagulopathy. These previous results warrant further preclinical and clinical studies in order to properly assess their safety and efficacy. While all the studies presented herein do present invasive methods of neuromodulation and hence represent a substantial barrier to be applied for trauma care, VNS and TNS could be applied clinically without much difficulty. There are promising noninvasive VNS and TNS clinical neuromodulation approaches which have similar beneficial effects as described with the invasive approaches, such as transcutaneous auricular VNS (Yu et al. 2017) and external TNS of the supratrochlear and supraorbital branches (Schoenen et al. 2013; DeGiorgio et al. 2011; Magis et al. 2013). Following more robust experimental testing, these noninvasive methods may be applied to HS in the future. We hope to see that these emerging therapies survive further scientific examination and develop into innovative clinical applications to serve the largely unmet needs of HS.

## Data Availability

Not applicable.

## Abbreviations

ANS: Autonomic nervous system
AP: Arterial pressure
EA: Electroacupuncture
HS: Hemorrhagic shock
PhNS: Phrenic nerve stimulation
PP: Perfusion pressure
TNS: Trigeminal nerve stimulation
VNS: Vagus nerve stimulation
